# GSPE Pre-Treatment Exerts Long-Lasting Preventive Effects against Aging-Induced Changes in the Colonic Enterohormone Profile of Female Rats

**DOI:** 10.3390/ijms24097807

**Published:** 2023-04-25

**Authors:** Alba Miguéns-Gómez, Marta Sierra-Cruz, M. Teresa Blay, Esther Rodríguez-Gallego, Raúl Beltrán-Debón, Ximena Terra, Montserrat Pinent, Anna Ardévol

**Affiliations:** 1 MoBioFood Research Group, Departament de Bioquímica i Biotecnologia, Universitat Rovira i Virgili, c/Marcel·lí Domingo n°1, 43007 Tarragona, Spain; albamiguensg@hotmail.com (A.M.-G.); msiecru@gmail.com (M.S.-C.); mteresa.blay@urv.cat (M.T.B.); esther.rodriguez@urv.cat (E.R.-G.); raul.beltran@urv.cat (R.B.-D.); ximena.terra@urv.cat (X.T.); anna.ardevol@urv.cat (A.A.); 2 Institut d’Investigació Sanitària Pere Virgili (IISPV), 43005 Tarragona, Spain

**Keywords:** procyanidins, intestine, aging, GLP-1, CCK

## Abstract

The impact that healthy aging can have on society has raised great interest in understanding aging mechanisms. However, the effects this biological process may have on the gastrointestinal tract (GIT) have not yet been fully described. Results in relation to changes observed in the enteroendocrine system along the GIT are controversial. Grape seed proanthocyanidin extracts (GSPE) have been shown to protect against several pathologies associated with aging. Based on previous results, we hypothesized that a GSPE pre-treatment could prevent the aging processes that affect the enteroendocrine system. To test this hypothesis, we treated 21-month-old female rats with GSPE for 10 days. Eleven weeks after the treatment, we analyzed the effects of GSPE by comparing these aged animals with young animals. Aging induced a greater endocrine response to stimulation in the upper GIT segments (cholecystokinin (CCK) and glucagon-like peptide-1 (GLP-1)), a decrease in the mRNA abundance of GLP-1, peptide YY (PYY) and chromogranin A (ChgA) in the colon, and an increase in colonic butyrate. GSPE-treated rats were protected against a decrease in enterohormone expression in the colon. This effect is not directly related to the abundance of microbiome or short-chain fatty acids (SCFA) at this location. GSPE may therefore be effective in preventing a decrease in the colonic abundance of enterohormone expression induced by aging.

## 1. Introduction

The gastrointestinal tract’s (GIT’s) main function is the digestion and absorption of nutrients, a key role in the body’s homeostasis and metabolism [1]. It is the first site of contact with the molecules we ingest and it acts as a physical and chemical barrier between the external and physiological environments. The GIT performs immune sensing, which protects the body from external risks [2]. Moreover, it oversees another relevant function, enteroendocrine activity, which is coordinated by enteroendocrine cells (EECs). These are specialized secretory cells scattered throughout the mucosal epithelium of the gut that constitute the largest endocrine organ in the body, even though EECs represent only 1% of the cells in the GI epithelium [3,4]. The intestinal microbiota exerts a relevant function in human health and disease. This population of microorganisms is involved in several processes such as nutrient extraction, metabolism and immunity [5]. Gut microbiota varies along the GIT, the colon being the richest area. Although it may vary due to age and external factors (medication use), in healthy conditions the microbiota is stable and shows a symbiotic interaction with the host.

Since life expectancy is increasing all over the world, knowledge on how to achieve healthy aging is becoming more important. Specifically, we need to understand the aging-related changes that occur in the GIT so that they may be prevented or delayed. Aging is a biological process characterized by the progressive functional decline of numerous interrelated physiological systems at multiple levels of the organism and resulting in reduced metabolic flexibility [6,7]. Several alterations of intestinal functions due to aging processes have been described, though some results are controversial. Reasons for the controversy may be the age of the subjects under study or comparisons between species. Balaskó et al. demonstrated the differential efficacy of intraperitoneally injected cholecystokinin (CCK) depending on age and found that the strongest resistance to the anorectic effect was observed in middle age (12-month-old Wistar males) [8]. Sandströ and El-Salhy showed that the number of CCK-producing cells in the duodenum of humans was lowest in a group of subjects aged 60–69 years old [9] but found no age-related differences in mice [10]. When MacIntosh et al. compared men aged 70 with men aged 27, they reported an age-related effect in CCK but not in glucagon-like peptide-1 (GLP-1) or peptide YY (PYY) secretion either at baseline or after stimulation in vivo [11]. Other authors have described changes in incretin profile [12,13], which could affect insulin release or impair the satiating effect of CCK [14]. Aging effects have also been defined at the gene expression level. The mRNA levels of *Pro-Gcg* showed a lower expression in the jejunum and the ileum of mouse models of aging [15]. Modifications have also been associated with the loss of gut microbial diversity in the aging population [16]. Among other changes, the phyla Bacteriodetes and Firmicutes remain dominant, though their relative proportions may vary with aging [17]. Curiously, centenarians typically do not experience these harmful changes, though their microbiota populations show marked differences from those of other elderly populations. This suggests that healthy microbiota may be a key to longevity [18].

Grape seed proanthocyanidin extracts (GSPE) have been shown to protect effectively against pathologies associated with aging. Among other benefits, these extracts are highly effective in improving the lipid [19,20] and glucose metabolism [21,22] and act as anti-inflammatory agents [23]. Atthe GIT, a dose of GSPE modulates enterohormone secretion, which has a satiating effect on food intake during treatment [24]. Treatment with GSPE improves the endocrine pancreas function and produces higher sensitivity to incretin hormones [25]. Moreover, some of these effects are maintained several months after the end of the treatment [26]. When we partially reproduced these results in 21-month-old female rats [27], we found the same satiating response as in young animals and observed that the treatment prevented some aging disruptions to metabolic parameters [27,28]. These results were obtained after oral administration of the GSPE. We therefore hypothesized that some beneficial effects of GSPE against several metabolic disturbances may be generated by its action at the GIT. 

In this paper we analyze the changes produced by aging in the enteroendocrine system of the GIT and the relationship between these changes and microbiota. We also analyze how GSPE treatment exerts its preventive anti-aging effects atthe GIT in 21-month-old female rats eleven weeks after GSPE administration. According to our previous results, this will support the long-lasting effect of this treatment [26,29]. 

## 2. Results

### 2.1. Differential Effects of Aging and GSPE Pre-Treatment on the Upper GIT Secretory Profile

Plasmatic total ghrelin at sacrifice and under fasting conditions was not modified by aging. However, the GSPE pre-treatment group tended to show lower levels of ghrelin (YOUNG: 1.38 ± 0.2; **AGED: 1.10 ± 0.1**; AGED GSPE: 0.93 ± 0.1 ^#^; ^#^ *p* ≤ 0.1 versus AGED). 

We also tested the functionality of the duodenum of the aged animals by working with duodenum explants to measure CCK secretion ex vivo. We found that, in agreement with the unmodified CCK plasmatic levels (Table 1), aging did not modify baseline CCK secretion (Table 1). To analyze tissue response to stimulation, we treated the ex vivo duodenum with meat peptone, a well-described enterohormone secretagogue. Our results showed that secreted CCK levels after peptone stimulation were significantly higher in AGED rats than in YOUNG rats (Table 1). The aged animals therefore showed a higher CCK response after stimulation. 

Compared with rats in the AGED group, those treated for ten days with GSPE showed lower baseline CCK secretion 11 weeks after the end of the treatment, which indicates the long-term effect of this treatment (Table 1). However, this effect was not reflected in the plasmatic levels of these animals (Table 1) and GSPE did not prevent the aging effect after peptone stimulation.

### 2.2. The Effects of Aging and GSPE on the GLP-1 System Differ between the Ileum and the Colon

**Figure 1:**
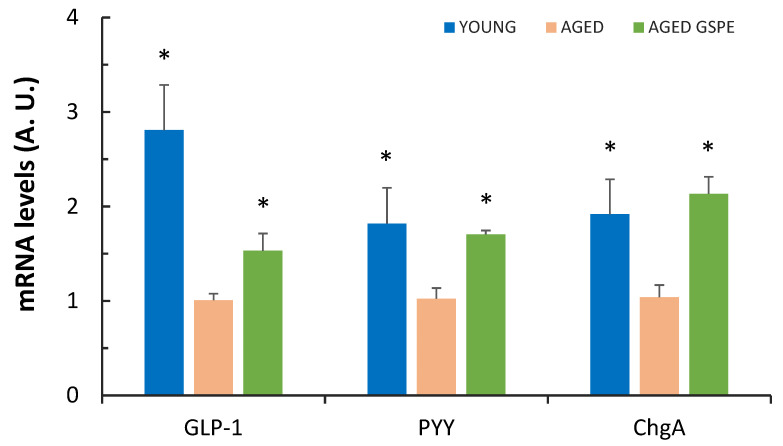
GLP-1, PYY and ChgA mRNA levels in colon. Values represent the mean ± SEM of four to six animals per group. A.U., arbitrary units. * Indicates *p* < 0.05 versus AGED, using Student’s *t* test.

The rats in the AGED group showed higher baseline active GLP-1 secretion in ileum explants (Table 2) (YOUNG: 31.76 ± 5.3; **AGED: 36.67 ± 11.8**; AGED GSPE: 16.6 ± 3.9; baseline aGLP-1 (pM)) but we did not observe this effect in colon explants (Figure 1). We cannot relate this baseline secretion of GLP-1 with changes in the expression levels of this hormone, since the mRNA levels showed no changes in the ileum and a lower mRNA expression in the colon. Ileum explants also showed a higher response to the peptone stimulation of aGLP-1 secretion in aged rats (Table 2). In this case, GSPE pre-treatment modified the stimulated GLP-1 secretion only in the ileum, thus causing a further increase (Table 2) and avoiding a decrease of GLP-1 mRNA levels in the colon (Figure 1).

Since aging suggested a higher baseline aGLP-1 secretion, which could lead to modified tissue sensitivity, we measured the expression of the GLP-1 receptor in the ileum and the pancreas. There were no changes due to aging in either of these tissues, nor were they significantly modified by GSPE pre-treatment, which suggests the absence of a long-term effect on the GLP-1 reception system in these organs (YOUNG: 1.20 ± 0.2; **AGED: 1.09 ± 0.2**; AGED GSPE: 1.21 ± 0.2 and YOUNG: 1.08 ± 0.2; **AGED: 1.06 ± 0.2**; AGED GSPE: 0.79 ± 0.1 for ileal and pancreatic GLP-1R mRNA levels, respectively).

### 2.3. GSPE Pre-Treatment Prevents an Aging-Induced Decrease in Colonic Enterohormone Expression

We have shown that the GLP-1 system was modified by GSPE differently in the ileum and the colon. On the other hand, the baseline secretion of PYY was not modified by aging or by GSPE pre-treatment in the ileum. This effect differed from that observed in the colon, where GSPE administered at the beginning of the study prevented a decrease in the mRNA expression of GLP-1, PYY and ChgA induced by aging (Figure 1). 

To find an explanation for the aging effects on colonic mRNA expression, we analyzed cecal SCFA content (Table 3). Butyric acid was the only SCFA analyzed that increased with aging, and GSPE pre-treatment did not prevent this increase. The only SCFA that was modified by GSPE pre-treatment was formic acid, which showed lower levels than in the rats of the AGED group. 

### 2.4. The Colonic Expression of GLP-1 Is the Most Relevant Factor for Discriminating between Groups

**Figure 2:**
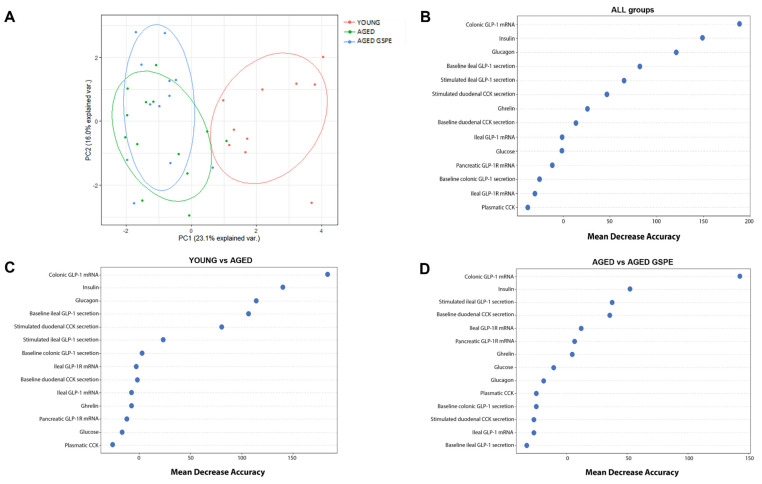
Multivariate analysis of enterohormone-related parameters along all the GIT. (**A**) Principal component analysis (PCA) of the parameters measured to test enterohormone system in the rats. Neither the PC1 nor the PC2 can separate the aged groups, but the PC1 clearly differentiates between the aged groups and the young group. (**B**–**D**) Mean decrease accuracy (MDA) from random forest analysis ranking the enteroendocrine variables according to their relevance in the classification of the groups. The variables are ordered top-to-bottom as most-to-least relevant in classifying the groups. (**B**) MDA considering all the three assayed treatments. (**C**) MDA for the comparison of YOUNG vs. AGED groups and (**D**) the comparison of AGED vs. AGED GSPE groups. Colonic GLP-1 mRNA, insulin and glucagon are the strongest variables in the separation of the groups, although glucagon does not affect the analysis when comparing the aged groups.

To better characterize our data, we ran PCA with all measurements of the enteroendocrine system of our animals. Figure 2A clearly shows that aging affects the enteroendocrine system, since we obtained two well-defined groups of young and aged rats. We then organized these variables according to their classification power using random forest analysis, first by considering all groups together and then by conducting the same analysis by pairs (Figure 2B). Our analysis of all three groups showed that the mRNA levels of GLP-1 in the colon had the highest classification power, followed by insulin and glucagon (mean decrease accuracy (MDA) > 100; (out-of-bag (OOB) error = 28.75%). When we used this multivariate analysis to compare groups by pairs, we also found that GLP-1 mRNA levels in the colon had the highest discriminatory power between aged and young animals (mean decrease accuracy (MDA) > 150; (out-of-bag (OOB) error = 8.70%). More interestingly, the GLP-1 mRNA level in the colon was also the variable that best explained the preventive effects of GSPE (mean decrease accuracy (MDA) > 100; out-of-bag (OOB) error = 32%).

**Figure 3:**
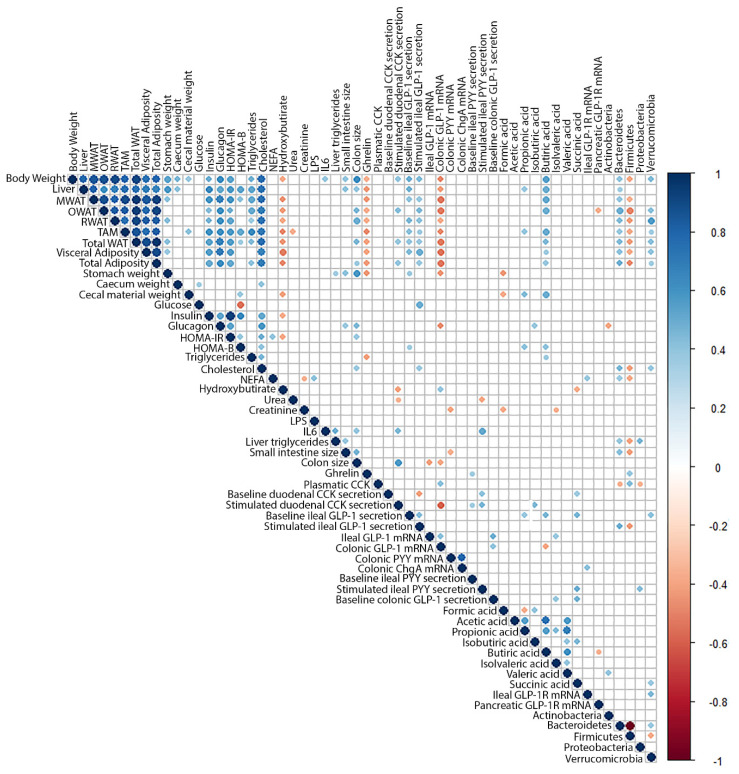
Correlation matrix between all the assayed parameters. All the parameters analyzed in this manuscript, and some previously published (or submitted) have been correlated. Scale color at right defines positive (blue) or negative (red) correlations between them. The intensity of the dot defines the strength of the statistically significant correlation (*p* < 0.05).

We completed our analysis with a Pearson’s correlation matrix (Figure 3) of the physiological and biochemical parameters of these animals [27] and Table S1, microbiota analysis (results submitted), and the current enterohormone data. Our results showed that plasmatic ghrelin was correlated negatively with most morphometric parameters (body, liver, white adipose tissue (WAT) and stomach weight), which indicates higher ghrelinemia in smaller animals. The plasmatic levels of this enterohormone also showed a positive correlation with Firmicutes phylum. The CCK levels in plasma also showed a positive correlation with Firmicutes and negative with Bacteroidetes and Verrucomicrobia phyla. 

Stimulated CCK secretion showed a positive correlation with ileal PYY secretions and colon size and cecal isobutyric acid content, but a negative correlation with colonic GLP-1 mRNA levels. Baseline ileal GLP-1 secretion also showed a positive correlation with most morphometric parameters (body, liver, stomach and retroperitoneal WAT weight) as well as with cecal butyric, succinic and propionic acids contents and Verrucomicrobia phylum. On the other hand, ileal GLP-1 mRNA correlated positively with GLP-1 mRNA levels and baseline secretion in colon, but negatively with colon size, which suggests a higher expression when the colon is smaller. Interestingly, stimulated ileal GLP-1 secretion showed a positive correlation with visceral adiposity, glycemia, and Bacteroidetes, which suggests an association with insulin resistance and a negative correlation with Firmicutes. GLP-1 receptor expression levels in the ileum showed a positive correlation with NEFA and Verrucomicrobia.

In the colon, there was no direct correlation between microbiota phyla and the parameters assayed. Baseline GLP-1 secretion correlated with the ileum parameters and positively with succinic and isovaleric acids. GLP-1 mRNA also showed a negative correlation with colon size, several morphometric parameters, butyric acid and glucagon but a positive correlation with urea and plasma CCK. 

We also found correlations with microbiota in upper enterohormones but not with colonic enterohormones. Only butyric and isovaleric cecal acids showed a correlation with colonic GLP-1.

## 3. Discussion

We have shown that the enteroendocrine systems of 25-month-old and 6-month-old female rats differed depending on the segment of the intestine analyzed. After stimulation, we found higher secretions of CCK in the duodenum and of GLP-1 in the ileum of aged animals, which agree with the previously described higher secretion of anorexic hormones in the elderly [13]. In these animals, we quantified food intake at the beginning of the experiment when the aged group were 21 months old and found no differences between the young and aged groups [27]. This may also have occurred at the end of the experiment but unfortunately no food intake data are available from that time point (though that result cannot be ruled out). This higher response to stimulation of GLP-1 in the ileum may also be related to the insulin resistance of these animals [27], which is usually associated with aging [30]. This ileal-stimulated GLP-1 secretion showed a positive correlation with visceral adiposity and glycemia, two typical parameters of insulin-resistance [31], and with Bacteroidetes phylum. GLP-1 receptor in the ileum showed a positive correlation with NEFA, which is also strongly linked to insulin resistance [32]. Ileum GLP-1 and GLP-1R parameters showed a positive correlation with Bacteriodetes and Verrucomicrobiota, respectively, though an increase of both phyla has been associated with an improvement in insulin resistance [33]. The involvement of Bacteriodetes in insulin resistance and their interaction with GLP-1 secretion has also been suggested by Hwang et al., who reported an improvement in insulin resistance linked to the lower presence of Bacteroidetes and Firmicutes associated with GLP-1 secretion [34]. Our data also therefore link GLP-1-stimulated secretion in the ileum with insulin resistance and microbiota restructuration in these animals, though the effect is contrary to that described by other authors [33].

The aging effect on GLP-1 mRNA levels showed no correlation with stimulated secretions. In the ileum we observed an inverse correlation between GLP1 expression and colon size, which suggests a compensatory mechanism that also occurs in the colon. Colonic GLP-1 mRNA also showed a negative correlation with butyric acid production in that part of the intestine. Our result differs from those of authors who have reported an effect of butyric acid in stimulating enterohormone cell formation [35]. Moreover, we found a lower expression of GLP-1, PYY and ChgA mRNA in the colon of animals that showed a higher butyric acid content, which suggests a contrary effect of butyric acid that limits the differentiation after chronic exposure that could be expected from our aging model. There is no direct correlation between the aging-induced changes of colonic mRNA levels and microbiota in these animals. In this study we also analyzed the preventive effect that GSPE pre-treatment may have on the effects of aging. We have shown that this treatment was not preventive in the ileum even though this segment had always shown sensitivity to GSPE pre-treatment in previous experiments. GSPE decreased the mRNA levels of enteroendocrine cells in the ileum of aged rats fed a chow diet and aged rats fed a cafeteria diet [36] but increased GLP-1 mRNA levels in the ileum of young female rats [37]. Where GSPE showed a preventive effect after 11 weeks of administration was in the colon. GSPE clearly prevented the decrease in GLP-1 mRNA levels caused by aging and had a similar effect on PYY and ChgA colonic mRNA expression, thus preventing a decrease in the expression levels of these enterohormones in aged animals. 

GSPE components easily reach the colon after being ingested in suitable quantities. Some of these compounds can be digested while others remain intact [38]. These phenolic structures can modify the microbiota population. A similar experiment to ours showed that a short administration of GSPE produced a decreased ratio of Firmicutes/Bacteroidetes in female rats [39] measured just after the end of the treatment. This effect now appears to be maintained for 11 weeks after the end of the treatment since our rats showed a similar profile (results submitted). However, this mechanism does not appear to be responsible for the observed GSPE preventive effects. Our correlation analysis did not show a direct relationship between colonic measurements and microbiota phyla. However, the changes in microbiota may participate indirectly since a lower Firmicutes/Bacteroides ratio has been correlated with higher butyrate production [40]. This could explain the correlation we found between butyric acid and GLP-1 colonic mRNA. In addition to these potential effects of GSPE in microbiota, GSPE can also act directly on enteroendocrine cells. Another mechanism could be the direct effect of GSPE in promoting L-cell differentiation in intestinal organoids, as we have shown previously [41], or the epigenetic effect of GSPE on enteroendocrine cells and precursors, as described by our group for the GLP-1 receptor [37].

Agonists of the GLP-1 receptor have been proven useful mainly in situations where there is a limited ability to produce GLP-1, as observed in some insulin-resistance situations and obesity [42]. This study was conducted in fasted animals, where it was not possible to measure blood active GLP-1. Our data showed how ageing has different effects on specific segments of the GIT and on the ability of a GSPE to exert long-term effects in the modulation of some parameters. Primary studies from our group in this research started by proving that GSPE acts as a GLP-1 stimulator. GSPE acutely administered increased GLP-1 secretion and limited food intake [24]. When we treated aged animals with the same GSPE treatment as in the present study, but simultaneously to the administration of a cafeteria diet, we also found an effect limiting food intake, reducing mesenteric adipose tissue and improving some insulin resistance indices [28]. Therefore, the results showed in this manuscript must be analyzed from the point of view of each segment of the GIT, and the identification of the different mechanisms altered by aging and targeted by GSPE along the GIT.

Summarizing, our study showed that aging affects the enterohormone system in the upper GIT, which favours a higher response to stimulation, an effect not prevented by GSPE. In the lower GIT, on the other hand, aging increases butyrate production and decreases the number of L-cells in the colon and mRNA levels of GLP-1 and PYY. This latter aging effect was prevented by GSPE pre-treatment even eleven weeks after the end of the treatment. GSPE may therefore be effective in preventing certain aging effects in the enteroendocrine system—especially in the area of the colon. 

## 4. Materials and Methods

### 4.1. Proanthocyanidin Extract

The grape seed extract rich in proanthocyanidins (GSPE) came from Les Dérivés Résiniques et Terpéniques (Dax, France). According to the manufacturer, the GSPE used in this study (lot 207100) had a total proanthocyanidin content of 76.9% consisting of a mixture of monomers of flavan-3-ols (23.1%), dimers (21.7%), trimers (21.6%), tetramers (22.2%) and pentamers (11.4%).

### 4.2. Animal Study

We used 34 female Wistar rats obtained from Envigo (Barcelona, Spain). Of these rats, 10 were two months old (210–220 g) and 24 were twenty-one months old (300–350 g). Treatment was performed as described by Grau-Bové et al. [27]. All procedures were approved by the Experimental Animal Ethics Committee of the autonomous government of Catalonia, Spain (Department of Territory and Sustainability, General Directorate for Environmental and Natural Policy, project authorization code: 10183). Briefly, the animals were housed individually at room temperature (23 °C) with a standard 12 h light–dark cycle and ad libitum access to tap water and a standard chow diet (2014 Teklad Global 14% protein rodent maintenance diet from Envigo, Barcelona, Spain). After a period of adaptation, the rats were weighed and divided into three experimental groups: YOUNG (two-month-old rats; n = 10); AGED (twenty-one-month-old rats; n = 12) and AGED GSPE (twenty-one-month-old rats receiving the GSPE pre-treatment; n = 12). GSPE was dissolved in tap water and orally gavaged to the AGED GSPE rats at a dose of 500 mg per kg BW (body weight) at 18.00, one hour before dusk. This dose has been chosen based on previous results from our group [43,44]. Rats in the YOUNG and AGED groups received an equivalent volume of tap water at the same time points. A chow diet was administered at dusk (19.00). Over the ten days of treatment, the three groups of rats began fasting at 15.00. To evaluate the long-term effects of GSPE, the animals were fed a chow diet for 75 more days (11 weeks) after the treatment. At the end of the study, the animals were euthanized by decapitation after a fasting period of 11 h. 

### 4.3. Blood and Tissue Collection

Blood was collected using lithium heparin (Deltalab, Barcelona, Spain) as an anticoagulant. Plasma was obtained by centrifugation (1500× *g* for 15 min at 4 °C) and stored at −80 °C in different aliquots until analysis. The samples to be analyzed for ghrelin were collected into a tube containing protease inhibitors (cOmplete ULTRA Protease Inhibitor Cocktail, Roche, NY, USA). The intestine and other tissues were carefully removed, measured and weighed. Different intestinal segments were excised from the duodenum, the ileum and the proximal colon. A sample of each segment was immediately frozen in liquid nitrogen and stored for mRNA analysis. Another piece of the duodenum, ileum and proximal colon were taken for the ex vivo study. The cecum was quickly weighed before and after the removal of cecal content. The cecal content was immediately frozen in liquid nitrogen and stored. All samples were stored at −80 °C until analysis. 

### 4.4. Ex Vivo Study

After the intestinal tube was washed with cold PBS buffer, the outer muscular layer was removed from the serosa layer with a scalpel. For each segment, the intestinal tube was then cut longitudinally and circular slices of tissue 5 mm in diameter were taken using a biopsy punch. The samples were kept at low temperature in an ice-cold Krebs–Ringer bicarbonate (KRB) buffer bath (Hepes 11.5 mM, CaCl_2_ 2.6 mM, MgCl_2_ 1.2 mM, KCl 5.5 mM, NaCl 138 mM, NaHCO_3_ 4.2 mM, NaH_2_PO_4_ 1.2 mM) supplemented with 10 mM D mannitol for the whole procedure. The tissue segments were placed in a prewarmed (37 °C) KRB-D-mannitol buffer for 15 min to stabilize the tissue. The medium was then replaced for the treatments: KRB-D- glucose buffer as a control and peptone from bovine meat, enzymatically digested (Cat. no: 70175, Sigma-Aldrich, Madrid, Spain), to stimulate enterohormone secretion. The tissue segments were incubated for 30 min in a humidified incubator at 37 °C, 95% O_2_ and 5% CO_2_. After this period, the whole volume was aliquoted and stored at −80 °C for further measurements. KRB containing 10 mM D-glucose was supplemented with protease inhibitors amastatin 10 µM (Enzo Life Sciences, Madrid, Spain), aprotinin 100 KIU (Sigma, Barcelona, Spain) and 0.1% fatty acid free-bovine serum albumin (BSA). 

#### Enterohormone Quantification

Plasma and tissue enterohormones were analyzed using commercial ELISA kits. Active GLP-1 (Catalogue no. EGLP-35K) and total ghrelin (Catalogue no. EZGRT-91K) kits were purchased from Millipore (Billerica, MA, USA). Total CCK (Catalogue no. EKE-069-04) and PYY (catalogue no. FEK-059-03) kits were purchased from Phoenix Pharmaceuticals (Burlingame, CA, USA).

### 4.5. Gene Expression Analysis

Total RNA and cDNA were obtained as previously defined [37]. Quantitative PCR amplification was performed using specific TaqMan probes from Applied Biosystems (Waltham, MA, USA): Rn01460420_g1 for PYY gene (*Pyy*), Rn00562293_m1 for proglucagon gene (*Gcg*), Rn00562406_m1 for GLP-1 receptor gene (*Glp1r*) and Rn00572200_m1 for chromogranin A gene (*Chga*). The relative expression of each gene was compared with the control group using the 2^−∆∆Ct^ method with PPIA (Rn00690933_m1) as a reference.

### 4.6. Quantification of Short-Chain Fatty Acids 

The concentration of short-chain fatty acids (SCFA) (formic, acetic, propionic, butyric, isovaleric, valeric, lactic and succinic acids) were assayed in cecal content thawed at 4 °C as previously described [36].

### 4.7. Statistical Analysis

Our results are presented as means ± SEM. We used Student’s *t*-test to analyze each treatment compared with the aged group. The Tukey (HSD) test was used as a post hoc test to identify differences between treatments. Multivariate statistics, i.e., principal component analysis (PCA), were also used. Random forest analysis was generated to identify the variables that made the largest contributions to the discrimination between groups. Finally, correlations between variables were assessed using the Pearson correlation coefficient. The statistical software we used comprised XLSTAT 2020.1 (Addinsoft, Barcelona, Spain), the R program, version 4.2.2 (http://cran.r-project.org) and SPSS 23.0 (IBM, Madrid, Spain). *p* values < 0.05 were considered statistically significant. 

## Figures and Tables

**Table 1:** Preventive effects of GSPE against aging on CCK secretion.

	YOUNG	AGED	AGED GSPE
CCK plasmatic levels (ng mL^−1^)	0.72 ± 0.05	**0.64 ± 0.03**	0.61 ± 0.03
Baseline CCK (ng mL^−1^)	0.23 ± 0.03	**0.30 ± 0.04**	0.18 ± 0.02 *
Stimulated CCK (ng mL^−1^)	0.50 ± 0.04 *	**0.72 ± 0.09**	0.64 ± 0.05

Baseline and stimulated levels were measured in the medium of the ex vivo experiment. Values represent mean ± SEM of seven to ten animals per group. * Indicates *p* values < 0.05 versus AGED, using Student’s *t* test. Bold letters highlight the reference group.

**Table 2:** Aging and GSPE effect on the ileum.

	YOUNG	AGED	AGED GSPE
Baseline aGLP-1 (pM)	59.4 ± 10.0 *	**156.5 ± 38.4**	135.5 ± 19.7
Stimulated aGLP-1 (pM)	196.7 ± 44.8 ^#^	**368.2 ± 82.4**	607.4 ± 90.2 ^#^
Baseline PYY (pg mL^−1^)	21.3 ± 5.7	**25.6 ± 4.9**	32.3 ± 1.2
Stimulated PYY (pg mL^−1^)	n.a.	**107.5 ± 15.7**	101.3 ± 7.4
GLP-1 mRNA (A. U.)	1.1 ± 0.2	**1.1 ± 0.2**	0.8 ± 0.1 ^#^

Values represent mean ± SEM of seven to ten animals per group. n.a., not analyzed. * Indicates *p* < 0.05. ^#^ Indicates *p* ≤ 0.1 versus AGED, using Student’s *t* test. Bold letters highlight the reference group.

**Table 3:** Levels of cecal SCFA content in the various groups.

	YOUNG	AGED	AGED GSPE
Formic	10.1 ± 1.6	**7.7 ± 1.2**	4.7 ± 1.3 ^#^
Acetic	25.6 ± 4.9	**26.6 ± 2.2**	28.0 ± 3.6
Propionic	5.2 ± 1.4	**7.6 ± 0.7**	6.5 ± 1.4
Isobutyric	0.3 ± 0.1	**0.5 ± 0.1**	0.4 ± 0.2
Butyric	4.5 ± 0.5 *	**9.1 ± 1.2**	6.7 ± 1.7
Isovaleric	0.3 ± 0.1	**0.4 ± 0.0**	0.3 ± 0.1
Valeric	0.5 ± 0.1	**0.6 ± 0.1**	0.5 ± 0.1
Lactic	0.0	**0.0**	0.0
Succinic	0.4 ± 0.2	**0.6 ± 0.2**	0.4 ± 0.1

The cecal content was collected and SCFA measured at sacrifice. Values represent the mean ± SEM of seven to ten animals per group (mmols g^−1^). * Indicates *p* ≤ 0.05 versus AGED, ^#^ indicates *p* ≤ 0.1 versus AGED, using Student’s *t* test. Bold letters highlight the reference group.

## Data Availability

Cora.rdr.

## Supplementary Materials

The following supporting information can be downloaded at: https://www.mdpi.com/article/10.3390/ijms24097807/s1.
