# Supplementary material for: GSPE Pre-Treatment Exerts Long-Lasting Preventive Effects against Aging-Induced Changes in the Colonic Enterohormone Profile of Female Rats
Source: Int J Mol Sci. 2023 Apr 25;24(9):7807. doi: 10.3390/ijms24097807 (PMC10177949; doi:10.3390/ijms24097807)
Supplement: Supplementary file 1 [file ijms-24-07807-s001.zip › ijms-2354513-supplementary.pdf]

Table S1. Supplementary material. Intestinal morphometric parameters at sacrifice.

	YOUNG	**21-MONTHS**	GSPE PRE
Small intestine (cm)	99.7 ± 1.7*	**106.9 ± 2.1**	108.3 ± 1.4
Small intestine (g)	5.9 ± 0.1*	**7.0 ± 0.2**	6.5 ± 0.2#
Ratio small intestine/BW (%)	2.3 ± 0.1*	**2.0 ± 0.1**	1.8 ± 0.1
Colon (cm)	1.2 ± 0.1*	**1.5 ± 0.1**	1.5 ± 0.1
Colon (g)	17.9 ± 0.5*	**20.1 ± 0.6**	20.5 ± 0.5
Ration colon/BW (%)	0.5 ± 0.0	**0.4 ± 0.0**	0.4 ± 0.0
Empty cecum (g)	0.7 ± 0.0#	**0.9 ± 0.1**	0.9 ± 0.1
Cecal material (g)	1.5 ± 0.2	**1.7 ± 0.2**	1.8 ± 0.4

BW, body weight. *Indicates $p \leq 0.05$. # Indicates $p \leq 0.1$ vs 21-MONTHS by Student's T test.
